# Closed duodenal loop syndrome in newborns: a rare case report of concomitant pyloric and distal duodenal atresias and focused literature review

**DOI:** 10.3389/fmed.2026.1930703

**Published:** 2026-09-07

**Authors:** Alessandro Boscarelli, Laura Travan, Tamara Stampalija, Jenny Bua, Leila Lo Bello, Elia Balestra, Anita Spezzacatene, Elena Sofia Marcandella, Jurgen Schleef

**Affiliations:** 1 Department of Pediatric Surgery and Urology, Institute of Maternal and Child Health - IRCCS “Burlo Garofolo”, Trieste, Italy; 2 Division of Neonatology, Institute for Maternal and Child Health - IRCCS Burlo Garofolo, Trieste, Italy; 3 Unit of Fetal Medicine and Prenatal Diagnosis, Institute for Maternal and Child Health IRCCS “Burlo Garofolo”, Trieste, Italy; 4 Department of Medicine, Surgery and Health Sciences, University of Trieste, Trieste, Italy; 5 Department of Radiology, Institute for Maternal and Child Health, IRCCS “Burlo Garofolo”, Trieste, Italy

**Keywords:** case report, closed duodenal loop, congenital pyloric atresia, duodenal atresia, neonatal intestinal obstruction

## Abstract

Congenital pyloric atresia (CPA) is a rare cause of neonatal gastric outlet obstruction. Although usually isolated, CPA may coexist with additional gastrointestinal atresias. Its association with distal duodenal atresia (DA) is particularly relevant because the segment between the two obstructions may become a closed duodenal loop into which biliary and pancreatic secretions continue to drain. We report the case of a male newborn delivered at 35 + 3 weeks of gestation after prenatal detection of severe polyhydramnios, recurrent bowel dilatation, abdominal effusion, and a cystic abdominal lesion. Postnatal radiography did not show the classic double-bubble sign. Exploratory laparotomy revealed CPA, marked dilatation of the second duodenal portion, distal DA, extensive adhesions, and jejunal perforations. Surgical management included adhesiolysis, reduction duodenoplasty, gastrointestinal continuity reconstruction, and jejunal perforations repair. The postoperative course was complicated by hemorrhagic–hypovolemic shock, coagulopathy, liver dysfunction, and the need for a second surgery due to increasing abdominal free fluid and suspected pneumoperitoneum, followed by progressive recovery. Follow-up demonstrated adequate gastric emptying and duodenal transit, satisfactory growth, and age-appropriate neurodevelopment. A focused narrative review on concomitant CPA and DA was also conducted. Concomitant CPA and distal DA should be considered when prenatal imaging shows a cystic abdominal lesion associated with polyhydramnios and ascites. Recognizing closed duodenal loop syndrome may improve prenatal counseling, atypical imaging interpretation, and surgical planning.

## 1 Introduction

Congenital gastrointestinal atresias are a heterogeneous group of developmental anomalies resulting in complete obstruction of the digestive tract. Their incidence varies according to the anatomical site involved. Duodenal atresia (DA) is among the more frequent causes of neonatal upper intestinal obstruction, whereas congenital pyloric atresia (CPA) is exceedingly rare, accounting for approximately 1% of intestinal atresias and occurring in about 1 in 100,000 live births (1–3).

CPA may occur as an isolated malformation or in association with other congenital anomalies. The best-described associations include epidermolysis bullosa, aplasia cutis congenita, and additional gastrointestinal atresias (2, 4, 5). Among gastrointestinal associations, concomitant pyloric atresia and DA are particularly uncommon but clinically important because the obstructions may involve both the proximal and distal duodenal limits.

Three anatomical variants of CPA are generally recognized: type I, consisting of a mucosal diaphragm or web; type II, characterized by a solid cord replacing the pyloric canal; and type III, represented by complete separation between the stomach and duodenum (2, 4). Surgical treatment depends on the anatomical type and associated lesions. In contrast with isolated CPA, cases associated with multiple intestinal atresias have historically carried a poor prognosis, particularly when occurring within the spectrum of hereditary multiple intestinal atresias or immunodeficiency-associated phenotypes (5, 6).

When CPA coexists with distal DA, biliary and pancreatic secretions may drain into an isolated duodenal segment trapped between the two obstructions. This creates a closed duodenal loop that can gradually dilate and, in some cases, lead prenatally to bowel perforation and subsequent fetal ascites or biliary peritonitis (6–11). Herein, we report the case of a newborn with concomitant CPA and distal DA presenting with recurrent fetal bowel dilatation, abdominal effusion, and suspected antenatal bowel injury. We also provide a narrative literature review of reports describing this rare association. A focused narrative literature search was conducted in PubMed/MEDLINE and Google Scholar, using the terms “pyloric atresia,” “duodenal atresia,” “pyloric atresia AND duodenal atresia,” “distal duodenal atresia,” and “closed duodenal loop.” The search included publications from database inception to August 2026, with no initial date restrictions. Only articles published in English and containing original, individually verifiable cases of concomitant congenital pyloric and duodenal atresias were included. Reference lists of eligible articles were also screened to identify additional reports; cases mentioned exclusively in secondary sources for which the original report could not be retrieved were excluded.

## 2 Case description

**Figure 1:**
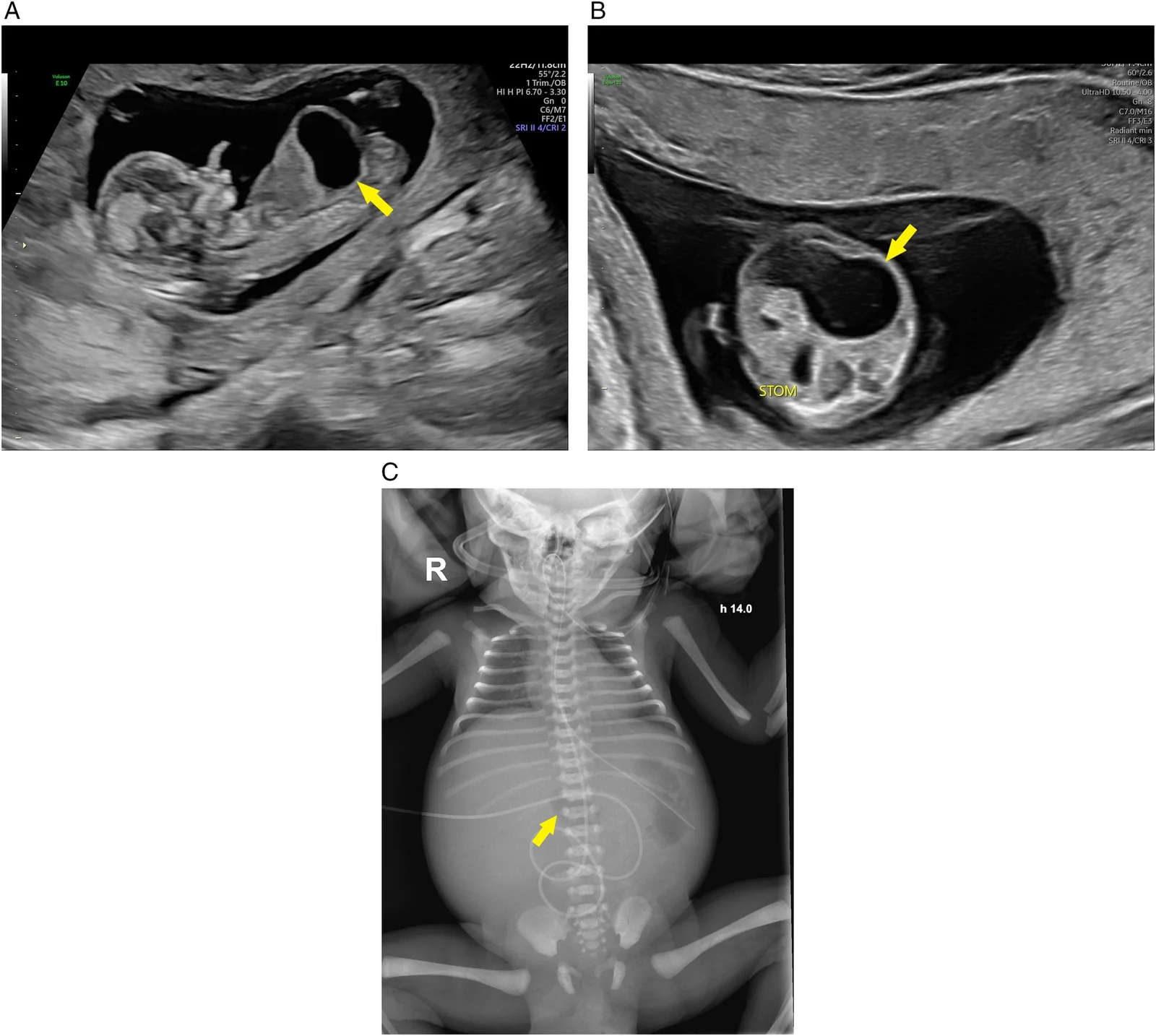
**(A)** Sagittal ultrasonography view of the fetus at 13 weeks of gestational age demonstrating a cystic lesion in the abdomen (*arrow*); **(B)** Axial ultrasonography view of the fetus confirming the presence of a cystic lesion (*arrow*) next to the stomach; **(C)** anteroposterior view of the x-ray at birth showing the stomach in the left upper quadrant and a single solitary bubble in the middle of the abdomen (*arrow*) highly suggestive of a closed duodenal loop syndrome.

A male infant was delivered through cesarean section at 35 + 3 weeks of gestation after a pregnancy complicated by progressive fetal intestinal abnormalities. Serial prenatal ultrasonography (US) revealed severe polyhydramnios, a cystic abdominal lesion initially thought to be suggestive of DA, which tended to be dilated and then normal and dilated again, and progressive abdominal effusion (Figures 1A,B). Two amnioreductions were required during the final weeks of pregnancy. Cesarean delivery was scheduled because of worsening bowel dilatation and breech presentation. Maternal history was notable for diet-controlled gestational diabetes mellitus. No relevant family history or infection-related risk factors were reported.

At birth, the infant presented Apgar scores of 8 and 10 at 1 and 5 min, respectively. Birth weight was 2,590 g. Mild respiratory distress required continuous positive airway pressure shortly after birth, with a maximum FiO_2_ level of 30%. Physical examination showed marked but soft abdominal distension and bilateral hydrocele, likely related to the previously documented fetal abdominal effusion. Initial laboratory evaluation revealed direct hyperbilirubinemia, elevated transaminase levels, and coagulation abnormalities. Notably, the cutaneous examination revealed no associated anomalies. Furthermore, a urine sample was also used to rule out the presence of congenital cytomegalovirus.

An abdominal US study immediately after birth confirmed a large cystic structure of approximately 36 × 28 millimeters in size located in the right upper abdomen, associated with dilated bowel loops and free intraperitoneal fluid. Plain abdominal radiography demonstrated diffuse abdominal opacity and two air-filled structures with air–fluid levels but did not show the classic double-bubble sign expected in isolated DA (Figure 1C).

**Figure 2:**
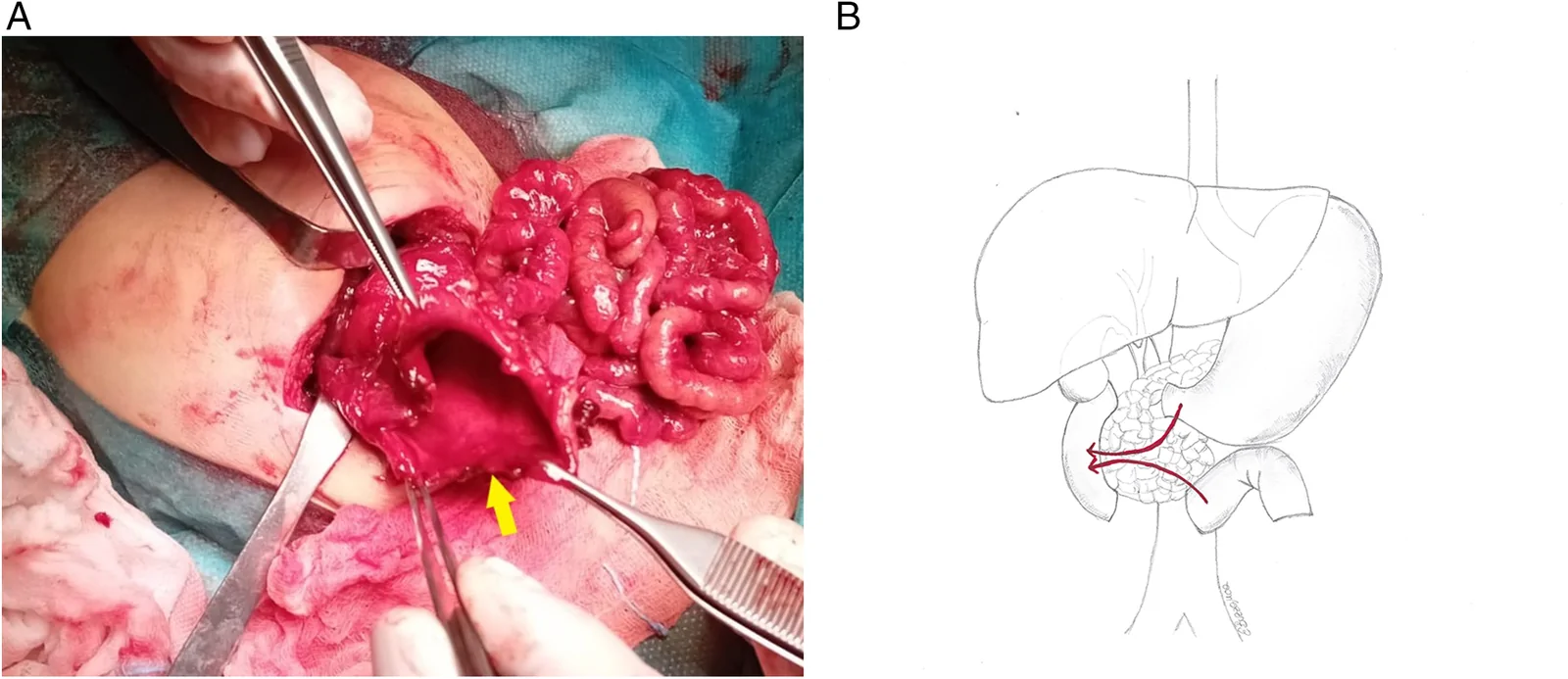
**(A)** Intraoperative view of the distended duodenal segment opened on the antimesenteric edge (*arrow*); **(B)** Handmade drawing illustrating the closed duodenal loop and the anastomoses with the stomach and the last portion of the duodenum (*red arrows*) performed to restore intestinal continuity and the biliary flow.

Exploratory laparotomy was performed on the second day of life through a transverse supraumbilical incision. Surgical exploration revealed extensive intra-abdominal adhesions, type III CPA, and marked dilatation of the second duodenal portion. A second obstructive lesion, consistent with type I DA, was identified between the second and third duodenal portions. Intestinal rotation was normal. The enlarged duodenal segment was incised on the antimesenteric wall and the intraoperative cholangiography permitted to distinctly identify the opening of the ampulla of Vater, thus confirming an isolated duodenal loop trapped between the pyloric atresia and a distal duodenal obstruction lying beyond the ampulla of Vater (Figure 2A). After adhesiolysis, reduction duodenoplasty was performed, and gastrointestinal continuity was restored by anastomosing the stomach, the dilated second duodenal portion and the last portion of the duodenum using absorbable sutures (Figure 2B). A transanastomotic nasojejunal tube was left in place at the end of the procedure. Finally, two jejunal perforations were also identified and treated via resection and primary end-to-end anastomosis with absorbable sutures.

The postoperative course was complicated by hemorrhagic–hypovolemic shock secondary to surgical blood loss and severe coagulopathy, requiring intensive hemodynamic support, blood product transfusions, and coagulation abnormality correction. Liver dysfunction worsened transiently during the early postoperative period. On postoperative day 11, increasing abdominal free fluid and pneumoperitoneum prompted a second surgical exploration. No bowel perforation was identified; the transanastomotic tube was removed, and an abdominal drain was placed. The infant's condition subsequently improved, with restoration of bowel function, normalization of laboratory parameters, and gradual resolution of previous abdominal findings.

During routine cranial US monitoring on day 23 of life, tetraventricular hydrocephalus was unexpectedly detected. Neurological findings remained normal, and serial imaging demonstrated stable ventricular enlargement without evidence of intraventricular hemorrhage. Brain magnetic resonance imaging confirmed mild ventriculomegaly associated with basal ganglia calcifications. Trio whole-exome sequencing did not identify pathogenic or likely pathogenic variants explaining the clinical phenotype.

**Figure 3:**
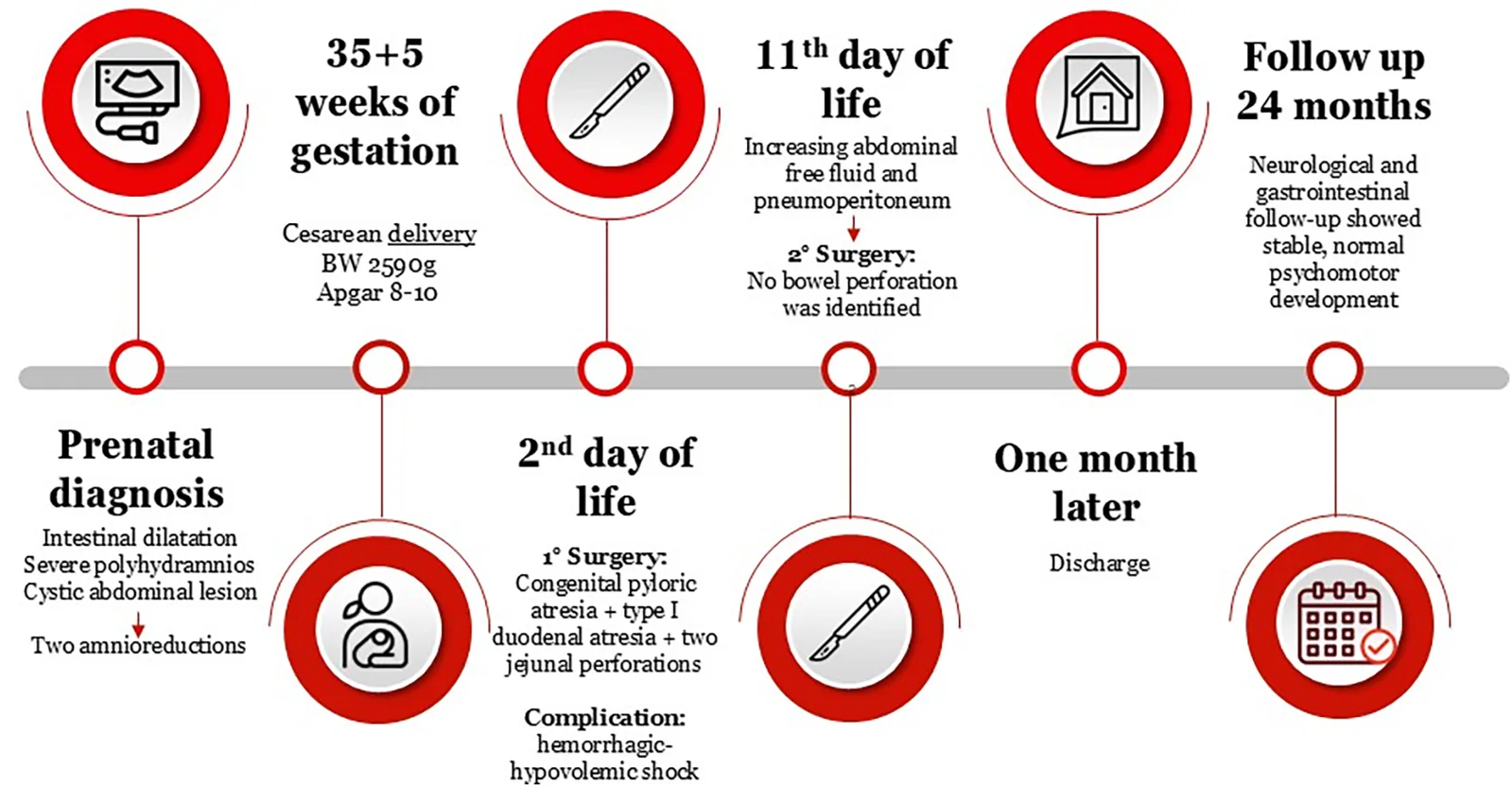
Case timeline chart.

The infant was discharged home at 50 days of life in good general condition. At 4 months, contrast gastrointestinal study demonstrated satisfactory gastric emptying and duodenal transit. After discharge, the infant was followed up in the Neonatal Follow-Up Clinic for high-risk newborns of our hospital. His most recent evaluation was performed at a corrected age of 16 months. Weight and height percentiles were between the 15th and 50th, while head circumference percentile was between the 85th and the 97th. Neuromotor development was appropriate for his corrected age according to the Touchpoints approach and the Brazelton scale. During the assessment, he was alert, attentive, and socially engaged, with appropriate interaction with the examiner. He walked independently with stable gait and good plantar support and was able to squat and stand up without assistance. He demonstrated age-appropriate fine motor skills, grasping and transferring objects between hands, manipulating toys appropriately, and engaging in simple cause-and-effect play. He was highly active, with a strong drive for movement, although his attention span was mildly inconsistent. Expressive language consisted of approximately four to five meaningful words. A timeline of the case is illustrated in Figure 3.

## 3 Discussion

The current literature on concomitant CPA and DA remains limited, consisting mostly of isolated reports and small case series (Table 1). Al-Salem and colleagues first reported CPA associated with intestinal atresia, contributing to the recognition that the combination of pyloric obstruction and distal intestinal obstruction may result in severe postoperative morbidity and mortality (6). Moreover, a subsequent report described CPA associated with DA, jejunal atresia, and intestinal duplication, which further expanded the spectrum of associated lesions (7). In 2000, Sarin reported pyloric atresia associated with multiple intestinal atresias and emphasized that, in several cases with concomitant DA, the duodenal obstruction was usually located distal to the ampulla of Vater. This anatomical arrangement creates a closed-loop obstruction into which bile and pancreatic secretions accumulate, causing massive distension and, in some cases, perforation or biliary peritonitis (8). Sencan et al. successively described a newborn with CPA, DA, jejunal atresia, apple-peel ileal atresia, and a pylorocholedochal fistula, confirming that CPA-DA may occur within a more complex spectrum of multiple intestinal anomalies (9). Additionally, in a series of congenital intrinsic duodenal obstruction published by Al-Salem, two patients presented with CPA associated with DA at the fourth duodenal portion. This combination created a closed duodenal loop, with pyloric atresia proximally and DA distally. Notably, only one patient developed duodenal perforation, and both of them died of sepsis in the context of immunodeficiency (10). The term “closed duodenal loop” was explicitly used by Al-Salem for the first time in 2007 to describe the anatomical configuration resulting from pyloric atresia associated with distal duodenal atresia, with accumulation of biliary and pancreatic secretions within the isolated segment; the same mechanism was later described by Kadian and Rattan in 2014 (10, 11). Specifically, Kadian and Rattan reported the case of a full-term male newborn with CPA and distal DA presenting as an abdominal lump. Computed tomography at birth demonstrated a massively dilated fluid-filled intestinal loop, and surgery confirmed pyloric atresia, DA at the duodenojejunal flexure, and ileal atresia. The infant deteriorated postoperatively with sepsis and was presumed to have died after leaving the hospital against medical advice (11). Overall, these reports support the concept that CPA with distal DA represents a distinct pathophysiological scenario rather than a simple coexistence of two congenital obstructions. The key determinant is the anatomical relationship between the distal duodenal obstruction and the ampulla of Vater. When the distal obstruction lies beyond the ampulla, biliary and pancreatic secretions enter a closed segment that cannot decompress proximally or distally, leading to progressive prenatal dilatation, cystic abdominal appearance, ascites, perforation, or biliary peritonitis (8, 10, 11).

**Table 1:** Pediatric cases of concomitant congenital pyloric and distal duodenal atresias reported in the current literature.

Author/s	N of patients	Prenatal features	Gestational age & Gender	Associated findings at birth	Outcome
Al-Salem et al. (6–10)	2	NR	NR	- CPA - Duodenal atresia (D4) - Multiple intestinal atresias	- Immunodeficiency - Both patients died of sepsis
Al-Salem (7)	1	NR	NR	- CPA - Duodenal atresia - Jejunal atresia - Intestinal duplication	NR
Sarin (8)	1	- Polyhydramnios - Double-bubble sign	- Full-term - Male newborn	- CPA - Duodenal atresia (D4) - Atresia of the ascending colon	- Septicemia and sclerema - Dead on postoperative day 13
Sencan et al. (9)	1	NR	- Full-term	- CPA - Duodenal atresia - Jejunal atresia - Apple-peel ileal atresia - Pylorocholedochal fistula	NR
Kadian and Rattan (11)	1	NR	- Full-term - Male newborn	- CPA - Duodenal atresia (D4) - Ileal atresia	- Postoperative sepsis - Presumed death after discharge against medical advice
Present case	1	- Severe polyhydramnios - Cystic abdominal lesion - Progressive fetal ascites	- 35 + 3 weeks - Male newborn	- CPA - Duodenal atresia (D2-D3) - Two jejunal perforations - Fetal ascites	- Survived - Favorable gastrointestinal and neurodevelopmental outcomes - 24-month follow-up

N, number; NR, not reported; CPA, congenital pyloric atresia.

CPA is among the rarest causes of neonatal gastrointestinal obstruction. Isolated CPA generally has a favorable prognosis after surgical correction. However, the outcome can be strongly influenced by associated anomalies, particularly multiple intestinal atresias, epidermolysis bullosa, aplasia cutis congenita, and immunodeficiency-associated phenotypes (2, 5, 12). The coexistence of CPA and DA is rare, with most available evidence derived from isolated reports or small series, as previously cited. This limited evidence makes it difficult to establish standardized recommendations for diagnosis, operative management, perioperative care, and follow-up.

Our case strongly supports the proposed mechanism underlying the so-called closed duodenal loop syndrome secondary to concomitant CPA and distal DA. Prenatal ultrasonography demonstrated progressive bowel dilatation, severe polyhydramnios, abdominal effusion, and a cystic abdominal lesion. Although these findings initially suggested isolated DA, postnatal imaging failed to show the classic double-bubble sign. This apparent discrepancy can be explained by concomitant pyloric atresia, which prevented gastric air from reaching the duodenum after birth. In retrospect, the prenatal cystic lesion most likely represented the massively dilated duodenal segment located between the pyloric and distal duodenal obstructions. In addition, the extensive adhesions encountered during surgery, together with jejunal perforations and abdominal effusion, suggest that bowel injury may already have occurred during fetal life. Chronic exposure of the peritoneal cavity to leaked intestinal, biliary, or inflammatory contents could explain both the marked adhesions and the unusually complex postoperative course. The presence of ascites, the combination of prenatal imaging evolution, and intraoperative findings strongly supports this interpretation.

Prenatal diagnosis of this condition remains challenging. In isolated CPA, the typical antenatal pattern includes polyhydramnios and gastric enlargement without distal bowel dilatation. By contrast, when CPA coexists with distal DA, imaging findings become more complex and may mimic duplication cysts, mesenteric cysts, or markedly dilated bowel loops. Awareness of the closed-loop mechanism may help clinicians interpret fetal abdominal cystic lesions, recurrent bowel dilatation, abdominal effusion, and progressive enlargement of an isolated fluid-filled structure as possible signs of concomitant pyloric and distal duodenal obstructions (12–15).

In patients with CPA associated with additional intestinal atresias, outcomes appear to be historically poor. Earlier reports frequently described death secondary to sepsis, intestinal failure, immunodeficiency, or extensive intestinal involvement-related complications. Some patients with multiple gastrointestinal atresias were later understood to fall within the spectrum of hereditary multiple intestinal atresias, a severe autosomal recessive disorder characterized by diffuse intestinal obstruction, immunological dysfunction, recurrent sepsis, and poor survival. Pathogenic *TTC7A* variants have been identified as a major genetic cause of this phenotype, linking severe intestinal malformations with combined immunodeficiency (12). In contrast with many previously reported patients, the present infant survived despite a severe postoperative course characterized by hemorrhagic–hypovolemic shock, coagulopathy, liver dysfunction, and reoperation. This favorable outcome was likely related to preservation of intestinal continuity, adequate bowel length, absence of an identified syndromic or immunodeficiency-associated genetic diagnosis, and intensive multidisciplinary management.

Notably, an additional unusual finding in our patient was postnatal hydrocephalus associated with basal ganglia calcifications. To our knowledge, this association has not been consistently reported in previous cases of concomitant CPA and DA. Infectious disease workups yielded negative findings, and trio whole-exome sequencing did not identify pathogenic or likely pathogenic variants explaining the phenotype. A direct relationship between the neurological and gastrointestinal findings therefore remains theoretical.

This review has some limitations. First, the rarity of CPA-DA restricts the available evidence to isolated case reports and small series. Second, several historical cases cited in older reviews could not be independently retrieved through contemporary databases and were therefore excluded from detailed synthesis. Third, many reports lack complete information regarding prenatal imaging, operative anatomy, immunological evaluation, genetic testing, and long-term follow-up. In addition, there are limitations of the case itself. In particular, the antenatal perforation could not be directly proven and a specific causal link between the neurological and gastrointestinal findings cannot be established.

In conclusion, concomitant CPA and distal DA are an exceptionally rare cause of neonatal intestinal obstruction. When the distal duodenal obstruction lies beyond the ampulla of Vater, the combination may create a closed duodenal loop, leading to progressive fetal bowel dilatation, cystic abdominal appearance, fetal ascites, perforation, and complex neonatal surgical management. Recognizing this mechanism is crucial for adequate prenatal counseling, interpretation of atypical imaging findings, operative planning, and even perioperative care.

## Data Availability

The original contributions presented in the study are included in the article/Supplementary Material, further inquiries can be directed to the corresponding author.
